# Effectiveness of exergaming interventions for adults with cancer: a systematic review

**DOI:** 10.3389/fonc.2026.1878533

**Published:** 2026-09-22

**Authors:** Jinqian Han, Mei Chan Chong, Wan Ling Lee, Enshe Jiang, Weizheng Zhang, Xin Liu

**Affiliations:** 1 Department of Bone and Soft Tissue, The Affiliated Cancer Hospital of Zhengzhou University, Henan Cancer Hospital, Zhengzhou, China; 2 Faculty of Nursing, Universiti Malaya, Kuala Lumpur, Malaysia; 3 School of Nursing, Faculty of Health Sciences, Nursing and Education, MAHSA University, Selangor, Malaysia; 4 Institute of Nursing and Health, Henan University, Kaifeng, China; 5 Department of Nursing, Faculty of Medicine, Universiti Kebangsaan Malaysia, Kuala Lumpur, Malaysia

**Keywords:** cancer, exergaming, physical function, psychological well-being, rehabilitation

## Abstract

**Background:**

Exergaming, which integrates physical exercise with interactive digital games, has gained attention as a supportive approach in cancer rehabilitation. However, uncertainty remains regarding its effects across different phases of cancer care and its feasibility in clinical and home-based settings. This systematic review synthesized evidence on the feasibility, safety, and effects of exergaming interventions among adults with cancer, with particular attention to interventions delivered during active treatment and after treatment completion.

**Methods:**

PubMed, Web of Science, the Cochrane Library, Scopus, Embase, CINAHL, and SPORTDiscus were searched from inception to April 2025. Randomized and nonrandomized intervention studies evaluating exergaming among adults with cancer were included. Outcomes were synthesized narratively across physical, psychological, symptom-related, quality-of-life, biological or physiological, and feasibility domains.

**Results:**

Twenty-five publications representing 19 distinct study cohorts met the inclusion criteria. Interventions varied substantially in platform, setting, duration, and training dose, most commonly lasting 4–16 weeks with 2–5 sessions per week. Reported recruitment rates ranged from 11.8% to 85.7%, adherence from 62% to 100%, and attrition from 0% to 47.4%. No serious intervention-related adverse events were reported among studies that provided safety data, although adverse-event reporting was incomplete. Favorable findings were reported for physical activity, mobility, balance, upper-extremity function, fatigue, and selected psychological and quality-of-life outcomes. In contrast, findings for muscle strength, pain, body composition, and biological or physiological markers were inconsistent.

**Conclusions:**

Exergaming appears to be a feasible and acceptable adjunct to cancer rehabilitation, with potential benefits for selected physical, symptom-related, and psychosocial outcomes. However, the evidence remains preliminary, and exergaming should be considered an adjunct rather than a replacement for conventional exercise or physiotherapy. Adequately powered randomized trials using standardized intervention protocols, consistent outcome reporting, longer follow-up, and more diverse cancer populations are needed.

**Systematic review registration:**

https://www.crd.york.ac.uk/prospero/display_record.php?ID=CRD42024530181, identifier CRD42024530181.

## Introduction

Cancer continues to pose a substantial global health burden, and advances in early detection and treatment have contributed to a steadily growing population of individuals living with and beyond cancer (1, 2). Despite improved survival, many patients experience persistent and often co-occurring symptoms that interfere with daily functioning and diminish overall quality of life (3). Cancer-related fatigue, pain, impaired physical function, and psychological distress are among the most commonly reported challenges and may continue for months or years following treatment completion (4–6). These symptoms arise from both the underlying disease and the cumulative effects of surgery, chemotherapy, and radiotherapy, yet they are frequently underrecognized and insufficiently managed in routine clinical care (7, 8). Increasing attention has therefore been directed toward rehabilitation approaches that can alleviate symptom burden and support long-term physical and psychological recovery in adults with cancer.

Regular physical activity is widely recognized as an effective nonpharmacological strategy for managing cancer-related symptoms and improving overall well-being (9, 10). Exercise interventions have demonstrated benefits across multiple outcomes, including improved functional capacity, reduced fatigue, and enhanced health-related quality of life (11, 12). However, adherence to recommended physical activity levels remains low among adults with cancer (13, 14). Treatment-related side effects such as fatigue, pain, and reduced mobility, together with psychological barriers including fear of movement and diminished motivation, frequently hinder participation in conventional exercise programs (15). Practical barriers, including transportation difficulties and limited access to trained oncology rehabilitation professionals, further restrict engagement (16–18). These challenges underscore the need for accessible and adaptable approaches that can promote sustained participation in physical activity. Exergaming, which integrates structured physical exercise within interactive digital environments, has therefore emerged as a potential strategy to enhance engagement in cancer rehabilitation by combining physical activity with real-time feedback and interactive elements (19). Exergaming interventions are commonly delivered through motion-sensing platforms such as Nintendo Wii and Xbox Kinect and may be implemented in hospitals, rehabilitation facilities, laboratories, or home settings (20). Home-based delivery may help reduce travel-related barriers and extend access to exercise support for patients who are unable to attend facility-based rehabilitation regularly (21). Remote monitoring and AI-supported coaching may also offer opportunities to track physical activity, provide individualized feedback, and support follow-up, although evidence for these approaches in cancer rehabilitation remains emerging (22).

Although interest in exergaming within oncology has increased, the current evidence base remains fragmented. Existing systematic reviews have typically focused on specific cancer types, such as breast cancer (23), or pediatric populations (24), or have examined selected outcomes such as fatigue, physical activity, or quality of life (20). As a result, the broader therapeutic impact of exergaming across physical, psychological, and symptom-related domains in adult cancer populations has not been fully synthesized. Moreover, prior reviews have seldom distinguished between interventions delivered during active treatment and those implemented after treatment completion, despite evidence that these phases are characterized by distinct symptom trajectories and rehabilitation needs. Feasibility outcomes, including recruitment, adherence, attrition, and safety, have also been inconsistently reported and rarely integrated into prior syntheses, limiting insight into the practical implementation of exergaming in clinical and home-based settings. In addition, emerging studies examining biological and physiological responses to exergaming have not yet been comprehensively reviewed (25, 26).

This systematic review aimed to evaluate the feasibility, safety, and effects of exergaming interventions for adults with cancer. Specifically, we synthesized evidence on physical, psychological, symptom-related, quality-of-life, and biological or physiological outcomes and examined findings according to whether interventions were delivered during active cancer treatment or after treatment completion.

## Methods

This systematic review was conducted and reported in accordance with the Preferred Reporting Items for Systematic Reviews and Meta-Analyses (PRISMA) guidelines (27) and was prospectively registered in PROSPERO (CRD42024530181).

### Search strategy

A comprehensive literature search was performed in PubMed, Web of Science, the Cochrane Library, Scopus, Embase, CINAHL, and SPORTDiscus from database inception to April 2025. The search strategy combined Medical Subject Headings (MeSH) and free-text terms related to cancer and exergaming. Key search terms included variations of “neoplasm, ” “cancer, ” and “tumor, ” combined with “exergaming, ” “active video game, ” and “virtual reality exercise.” Reference lists of all included studies were manually screened to identify additional eligible articles. The complete search strategies are presented in **Supplementary Table S1**.

### Eligibility criteria

Studies were eligible for inclusion if they met the following criteria: (1) the study population included adults aged 18 years or older with any cancer diagnosis, either during active treatment or after treatment completion; studies including healthy comparison participants were eligible when outcomes for participants with cancer could be identified separately; (2) the intervention involved exergaming that required structured bodily movement or physical activity delivered through an interactive digital platform; (3) the study employed a randomized controlled, nonrandomized comparative, single-group feasibility, or pilot design; and (4) at least one physical, psychological, symptom-related, quality-of-life, biological or physiological, feasibility, or safety outcome was reported.

Nonprimary research articles, conference abstracts, reviews, and study protocols were excluded. Crossover trials were not included because potential carryover effects and insufficient washout periods may compromise the interpretation of intervention effects, particularly in populations undergoing cancer treatment or recovery.

### Screening process

All records identified through the database searches were imported into a reference management system, and duplicate records were removed. Two reviewers independently screened the titles and abstracts according to the predefined eligibility criteria. Records were excluded at this stage if they did not meet the specified population, intervention, study design, or outcome criteria. Full-text articles were subsequently assessed for eligibility, and the specific reasons for exclusion at the full-text stage are presented in the PRISMA flow diagram. Disagreements were resolved through discussion, with consultation of a third reviewer when necessary.

### Data extraction and synthesis

Two reviewers independently extracted data using a standardized extraction form. Discrepancies were resolved through discussion or consultation with a third reviewer. Extracted information included study design, country, sample size, participant characteristics, cancer type and treatment stage, exergaming platform, intervention intensity, frequency and duration, intervention timing, delivery setting and provider, comparison conditions, and reported outcomes. Where available, effect estimates, 95% confidence intervals, and standardized effect-size measures reported by the original studies were also extracted. Multiple publications arising from the same or overlapping participant cohorts were identified by comparing study settings, recruitment periods, sample characteristics, intervention descriptions, and author information. These publications were treated as separate reports but were counted as a single study cohort, and overlapping participants were not counted more than once in the total sample size.

Outcome domains were predefined to ensure consistency between data extraction and result presentation. Feasibility and safety outcomes included recruitment, adherence, attrition, and adverse events. Physical outcomes included balance, muscle strength, mobility, range of motion, habitual physical activity, and functional performance. Other outcome domains included symptom-related measures, such as fatigue and pain; psychological measures, such as anxiety, depression, mood, and self-efficacy; and quality of life. Biological or physiological outcomes, including biochemical markers, body-composition measures, and clinical indicators, were extracted and reported separately when available.

A narrative synthesis was conducted because of substantial clinical and methodological heterogeneity across the included studies. Specifically, the studies differed in design, cancer type and treatment stage, participant characteristics, exergaming platforms, intervention frequency and duration, comparison conditions, and the instruments used to assess outcomes. These differences limited the comparability of the findings and made statistical pooling inappropriate. Results were therefore synthesized by outcome domain, including feasibility and safety, physical outcomes, psychological outcomes and quality of life, symptom-related outcomes, and biological or physiological outcomes.

### Quality assessment

Methodological quality was assessed using appraisal tools appropriate to each study design. Randomized controlled trials were evaluated using the Cochrane Risk of Bias 2 (RoB 2) tool (28). Nonrandomized comparative studies were assessed with the Risk of Bias in Non-randomized Studies of Interventions (ROBINS-I) tool (29), while single-group feasibility studies were appraised using the non-comparative Methodological Index for Non-Randomized Studies (MINORS) criteria (30). All quality assessments were conducted independently by two reviewers. Any disagreements were resolved through discussion, with consultation of a third reviewer when consensus could not be reached.

## Results

### Study selection

The study selection process is presented in the PRISMA flow diagram (**Figure 1**). A total of 2, 493 records were identified across seven databases. After the removal of 961 duplicates, 1, 532 records were screened based on their titles and abstracts, and 50 reports were assessed for full-text eligibility. Twenty-five publications representing 19 distinct study cohorts met the inclusion criteria and were included in the final synthesis.

**Figure 1:**
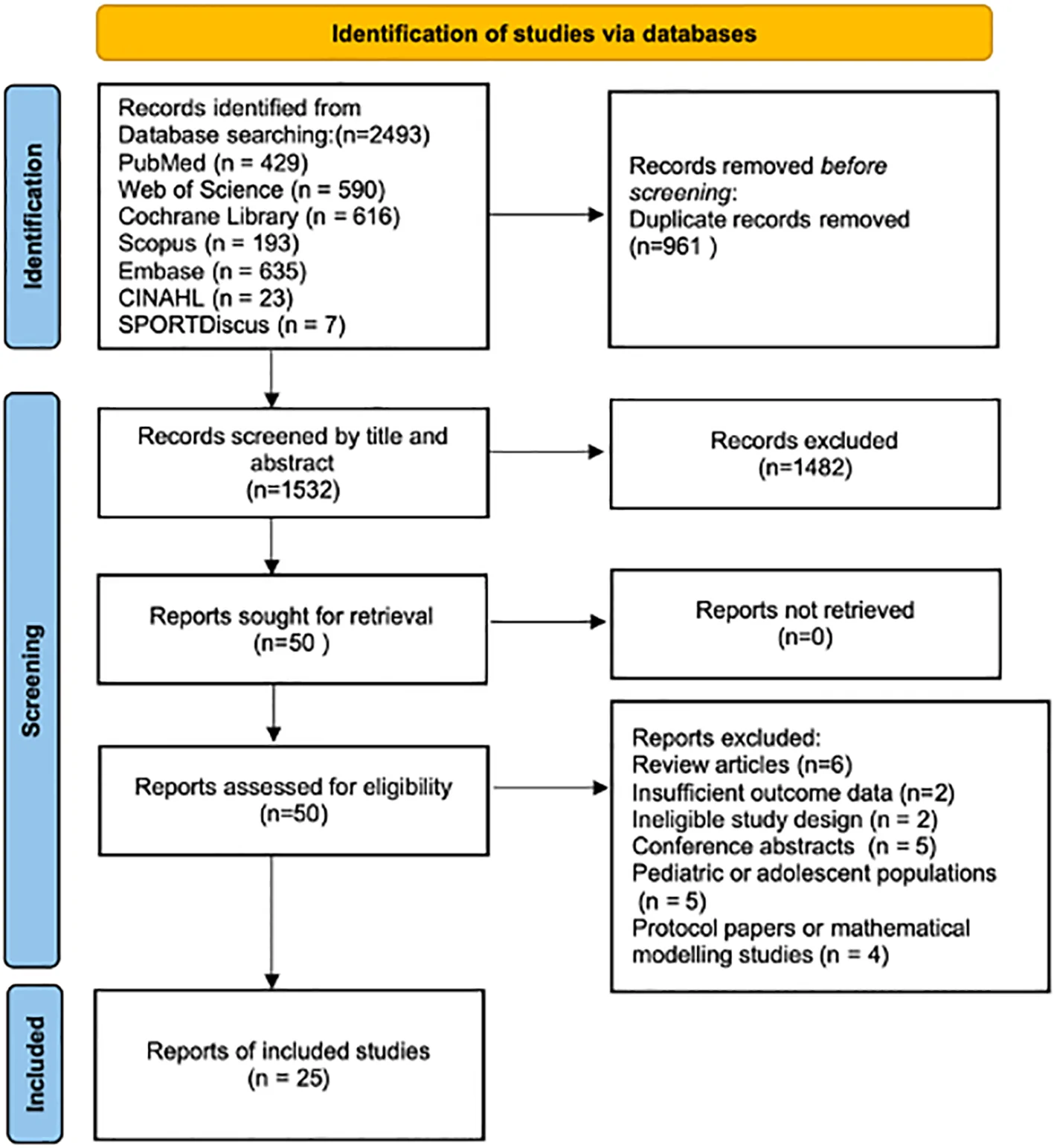
PRISMA flow diagram.

### Study characteristics

The main characteristics of the included studies are summarized in **Table 1**. Across the 19 study cohorts, 704 participants were included, comprising 689 adults with cancer and 15 healthy comparison participants. Participant ages ranged from 21 to 81 years. Breast cancer was the most frequently studied diagnosis (33, 42, 43, 45–50), followed by prostate cancer (26, 34, 44), lung cancer (31, 32), hematologic malignancies (35, 38), head and neck cancer (41), and mixed or other cancer types (25, 36, 37, 39, 40, 51–53).Cancer stage ranged from stage 0 to stage IV, although staging information was not consistently reported.

**Table 1:** Characteristics of included studies.

Authors and Year	Country	Study design	Cancer type (stage)	Sample size and age, years	Exergaming platform and games	Intervention protocol (intensity, frequency, duration)	Intervention timing	Setting / provider	Comparator
Hoffman et al. (31, 32) (2013, 2014)	USA	Feasibility study	Non-small cell lung cancer (stages I–III)	EG: n = 7; 64.6 ± 6.5 (range 53–73)	Nintendo Wii Fit Plus (downhill skiing, soccer, and golf)	Light intensity; 5 sessions/week; started at 5 min/session and increased by 5 min when the prespecified progression criterion (>70%) was met; 16 weeks	Initiated before surgery and continued after surgery	Home-based / nurse	No control group
House et al. (33) (2016)	USA	Feasibility study	Breast cancer (stage NR)	EG: n = 12; 57.8 (range 22–78)	BrightArm Duo Rehabilitation System (Breakout 3D, Card Island, Remember That Card, Musical Drums, Xylophone, and Pick & Place)	Table tilt progressively increased from 0° to 20°; 2 sessions/week; 20–50 min/session; 8 weeks	Approximately 9.5 years after surgery	Laboratory / occupational therapist	No control group
Sajid et al. (34) (2016)	USA	Randomized three-arm pilot trial	Prostate cancer (stage NR)	EG: n = 8, 77.5 ± 6.7; SPTG: n = 6, 75.7 ± 9.5; CG: n = 5, 71.8 ± 5.0	Nintendo Wii Fit (games NR)	Moderate intensity; 5 sessions/week; 45 min/session; 12 weeks	≥3 months after initiation of ADT	Home-based / exercise physiologist	SPTG: supervised walking and resistance-band training (RPE 3–5; ≥5 sessions/week); CG: usual care
Tsuda et al. (35) (2016)	Japan	Feasibility study	Hematologic cancer (stage NR)	EG: n = 16; 66 (range 60–76)	Nintendo Wii Fit with Balance Board (Hula Hoop and Basic Step)	Light-to-moderate intensity; 5 sessions/week; approximately 20 min/session; continued until discharge (median 23.5 days)	During active treatment	Hospital / physiotherapist	No control group
Silva Alves et al. (25, 36, 37) (2017, 2018, 2020)	Brazil	Quasi-experimental controlled study	Various cancers, including gastrointestinal, breast, abdominal, pelvic, and oropharyngeal cancers (stage NR)	CRG: n = 15, 57.13 ± 16.74; CAG: n = 15, 63.29 ± 7.34; CG: n = 15, 56.73 ± 11.94	Xbox 360 Kinect (Your Shape Fitness Evolved 2012; Wall Breaker, Stomp It, and Run the World)	Intensity NR; 2–3 sessions/week (mean: CRG 2.38, CAG 2.41, CG 2.47); 20 sessions over 8–12 weeks	CRG: during chemotherapy and/or radiotherapy; CAG: ≥6 months after treatment completion; CG: healthy participants	Laboratory / researcher	Comparisons among the active-treatment, post-treatment, and healthy groups; all groups received the same exergaming intervention
Schumacheret al. (38) (2018)	Germany	Randomized controlled trial	Hematologic cancer (stage NR)	EG: n = 19, 56 (range 21–65); SPTG: n = 23, 56.5 (range 23–69)	Nintendo Wii (Wii Sports, Wii Fit, and Balance Board)	Intensity NR; 5 sessions/week; 30–45 min/session; 4 weeks	Initiated ≥3 days before HSCT and continued during transplantation and inpatient recovery	Hospital / physiotherapist	Supervised endurance and resistance training, including bed-based exercises
Furtado et al. (39, 40) (2018, 2020)	Brazil	Quasi-experimental controlled study	Various cancers, including gastrointestinal, breast, abdominal, pelvic, ovarian, prostate, and oropharyngeal cancers (stages I–III)	EG: n = 19, 61.46 ± 8.79; CG: n = 19, 57.62 ± 7.57	Xbox 360 Kinect (Your Shape Fitness Evolved)	Intensity NR; 2–3 sessions/week; up to 50 min/session; 8–10 weeks	During active cancer treatment	Laboratory / physiotherapist	Conventional care without exergaming
Wang et al. (41) (2018)	USA	Feasibility Study	Head and neck cancer (Stage II-IV)	EG: n = 8; 57.6 ± 13.3	Nintendo Wii Fit (Aerobics, strength training, flexibility training (yoga), and balance)	Moderate intensity; progressed to 30 min/day, 5 days/week, with strength training twice weekly; one 1-hour home visit per week; 6 weeks	Within a month after cancer treatment (chemotherapy, radiation, or chemoradiation)	Home-based / nurse	No control group
Atef et al. (42) (2020)	Egypt	Quasi-randomized comparative study	Breast cancer-related lymphedema (stages I–II)	EG: n = 18, 54.07 ± 8.28; CG: n = 18, 53.07 ± 7.24	Nintendo Wii (tennis, triceps extension, and rhythmic boxing)	Intensity NR; 2 sessions/week; 30 min/session; 4 weeks	≥6 months after surgery	Hospital / physiotherapist	Conventional lymphedema rehabilitation, including pneumatic compression, manual lymphatic drainage, a home program, and education
Feyzioğlu et al. (43)(2020)	Turkey	Randomized controlled trial	Breast cancer (stage NR)	EG: n = 19, 50.84 ± 8.53; CG: n = 17, 51.00 ± 7.06	Xbox 360 Kinect (darts, bowling, boxing, beach volleyball, table tennis, and Fruit Ninja)	Intensity NR; 2 sessions/week; 45 min/session; 8 weeks	Early postoperative phase	Hospital / physiotherapist	Standard physical therapy; both groups also walked for 30 min three times/week
Villumsen et al. (26, 44) (2019, 2024)	Denmark	Randomized Controlled Trial	Prostate cancer (stages T1c–T3c)	EG: n = 23, 67.6 ± 4.6; CG: n = 23, 69.8 ± 4.4	Xbox Kinect (Your Shape Fitness Evolved, Sports, and Adventures)	Games selected to increase heart rate; 3 sessions/week; 60 min/session; 12 weeks	During continuous ADT	Hospital / physiotherapist	Usual care
Martínez de Albéniz Santamaría et al. (45) (2021)	Spain	Single-group pre–post study	Breast cancer-related lymphedema (stage NR)	EG: n = 12; 58.27 ± 12.34	Wii Sports and Wii Fit Plus (bowling, golf, Step, River Downstream, Fishing Under Zero)	Intensity NR; 1 session/week; 30 min/session; 5 weeks	Diagnosed with lymphedema secondary to breast cancer	Hospital / therapist	No control group
Basha et al. (46) (2022)	Egypt	Single-blind randomized controlled trial	Breast cancer-related lymphedema (stages I–III)	EG: n = 30, 48.83 ± 7.00; CG: n = 30, 52.07 ± 7.48	Xbox Kinect (darts, bowling, boxing, table tennis, Fruit Ninja, and beach volleyball)	Intensity adjusted to participant performance; 5 sessions/week; 1 session/day; 8 weeks	≥1 year after diagnosis	Outpatient center / physiotherapist	Resistance exercise; both groups received complex decongestive physiotherapy
Park et al (47). (2023)	Korea	Randomized controlled trial	Breast cancer (stage NR)	EG: n = 50, 42.56 ± 9.06; CG: n = 50, 47.30 ± 8.55	Xbox One Kinect for Windows (motion-tracking rehabilitation exercises involving 25 upper- and lower-extremity joints)	Progression individualized according to performance during the first 4 weeks; daily exercise; 8 weeks	After axillary lymph node dissection or breast reconstruction following mastectomy	Online / physician	Brochure-based home rehabilitation
Feyzioğlu et al. (48) (2022)	Turkey	Pilot study	Breast cancer (stages II–III)	EG: n = 15; 50.13 ± 8.79	Xbox 360 Kinect (Beach Volleyball, Bowling, Boxing, and Fruit Ninja)	Intensity NR; frequency NR; 5-min warm-up/adaptation followed by 30 min of supervised exergaming; 12 weeks	At 12 weeks after unilateral mastectomy	Hospital / physiotherapist	No control group
Swartz et al. (49)(2022)	USA	Randomized pilot trial	Breast cancer (stages 0–IV)	EG: n = 30, 56.10 ± 10.65; CG: n = 30, 58.67 ± 10.33	Wii Fit U and Xbox 360 Kinect (Just Dance, Zumba, Your Shape Fitness Evolved 2012, and other dance/fitness games)	Initially low intensity, progressing toward more frequent selection of fitness-based activities; frequency NR; 60 min/session; 13 weeks	After breast cancer diagnosis; exact treatment phase NR	Conference room / facilitator	Standard care
Swartz et al. (50)(2023)	USA	Randomized pilot and feasibility trial	Breast cancer (stages 0–III)	EG: n = 10, 64.90 ± 8.03; CG: n = 10, 62.60 ± 4.20	Xbox 360 Kinect (Zen Energy, yoga, kickboxing, upper- and lower-body training, Zumba, and other fitness games)	Intensity individualized according to participant interests and abilities; 1 session/week; 60 min/session; 12 weeks	After primary breast cancer diagnosis; treatment phase NR	Online / therapist	Phone-based survivorship discussions plus Mi Band self-monitoring
Schrempf et al. (51)(2023)	Germany	Randomized controlled trial	Colorectal cancer (stages I–IV)	EG: n = 31, 60.4 ± 9.5; CG: n = 31, 60.9 ± 9.7	Holofit app on Oculus Quest 2 with handheld controllers (virtual rowing or cycling)	Moderate intensity, progressing to ≤75% of maximum heart rate; weekdays; up to 30 min/session; continued until discharge	From the first postoperative day	Hospital / provider NR	Standard physiotherapy
Alves et al. (52, 53) (2024, 2025)	Portugal	Parallel-group randomized controlled trial; related quasi-experimental report	Intestinal, gastric, and other cancers (stage NR)	EG: n = 35, 63.1 (range 30–78); CG: n = 35, 62.3 (range 39–81)	Nintendo Wii (Basic Step, Penguin Slide, Super Hula Hoop, Bird’s-Eye, and Bull’s-Eye)	Intensity NR; sessions delivered on postoperative days 2–7; 15 min/day on days 2–3, 20 min/day on days 4–5, and 30 min/day on days 6–7; 1 week	From postoperative day 2	Hospital / provider NR	Usual care

Values are presented as mean ± SD unless otherwise indicated. Multiple publications derived from the same or overlapping study cohort are presented in a single row, and participants were counted only once in the total sample. ADT, androgen deprivation therapy; CAG, cancer aftercare group; CG, control/comparison group; CRG, chemotherapy and/or radiotherapy group; EG, exergaming group; HSCT, hematopoietic stem cell transplantation; NR, not reported; RPE, rating of perceived exertion; SPTG, supervised physical training group..

The studies were conducted across North America, South America, Europe, and Asia, with the largest numbers of publications originating from the United States (31–34, 41, 49, 50), Brazil (25, 36, 37, 39, 40), Germany (38, 51), Egypt (42, 46), Portugal (52, 53), Turkey (43, 48), and Denmark (26, 44). Multiple publications were derived from the same or overlapping participant cohorts, including those by Hoffman et al. (31, 32), Silva Alves et al. (25, 36, 37), Furtado et al. (39, 40), Villumsen et al. (26, 44), and Alves et al. (52, 53). By contrast, the studies by Swartz et al. (49, 50) and Feyzioğlu et al. (43, 48) implemented interventions at different time points and were analyzed separately.

### Intervention characteristics

Across studies, exergaming interventions were delivered using a range of platforms, including Nintendo Wii (31, 32, 34, 35, 38, 41, 42, 45, 52, 53), Xbox Kinect (25, 26, 36, 37, 39, 40, 43, 44, 46–48), one combining both systems (49), and other specialized systems included the BrightArm Duo Rehabilitation System (33) and the Holofit application delivered through Oculus Quest 2 (51). Intervention intensity ranged from light (31, 32) to moderate (34, 41), with seven studies incorporating progressive or adaptive difficulty (26, 44, 46, 47, 49–51). Some interventions incorporated progressive or individually adapted difficulty according to participant performance. Intervention frequency varied from one to seven sessions per week, most commonly two to five sessions weekly. Session duration ranged from 5 to 90 minutes, and most interventions lasted between 8 and 12 weeks. However, shorter inpatient interventions and longer home-based programs were also reported.

Interventions were delivered at different stages of cancer care. Some began before surgery and continued during postoperative recovery (31, 32), long-term post-surgery (33), during active treatment (25, 35–37, 39–41, 49, 50), post–androgen deprivation therapy initiation (26, 34, 44), pre–hematopoietic stem cell transplantation (38), post-breast surgery (42, 43, 47, 48), and two studies specifically targeted patients with breast cancer–related lymphedema (45, 46), and two focused on early postoperative rehabilitation within the first postoperative days (51–53).

Delivery settings included hospitals (26, 35, 38, 42–45, 48, 51–53), laboratories (25, 33, 36, 37, 39, 40, 49), home-based environments (31, 32, 34, 41), outpatient centers (46), and online or hybrid formats (47, 50). Most interventions were supervised by physiotherapists (26, 35, 36, 38–40, 43, 44, 46, 48), therapists (33, 45, 50), Physicians (47), nurses (31, 32, 41), research staff (25, 36, 37), trained facilitators (49), or an exercise physiologist (34), while three studies did not specify the provider (51–53). Comparator groups received usual care, no additional intervention, conventional rehabilitation, or structured exercise programs.

### Feasibility and safety

Recruitment outcomes were reported in the nineteen studies (25, 33–43, 46, 48–53). Among studies reporting recruitment as a percentage, rates ranged from 11.8% to 85.7%. The lowest recruitment rate was reported in a home-based intervention for patients with head and neck cancer (41), whereas the highest was reported among patients undergoing hematopoietic stem cell transplantation (38). Adherence ranged from 62% to 100% in 11 studies (31–33, 35, 41, 48–53), with three studies reporting full adherence (48, 52, 53). Attrition rates varied between 0% and 47.4%. Reported reasons for withdrawal included disease progression, treatment-related deterioration, medical complications, logistical or scheduling difficulties, hurricane-related disruption, and death (33–35, 38, 39, 41). Four studies employed standardized screening (KPS) (31, 32, 41), ECOG (41), MET (51) to assess exercise readiness. Safety monitoring in eight studies identified no intervention-related adverse events (26, 31, 32, 35, 41, 44, 47, 51). Reported musculoskeletal complications did not exceed CTCAE Grade 2 (35, 41), and unmanaged symptoms were attributed to cancer treatment rather than exergaming (31, 32).

### Methodological quality

Methodological quality varied across study designs (**Tables 2**–**4**). Among the 10 randomized study cohorts assessed using RoB 2, nine were judged to have some concerns regarding overall risk of bias, while one was rated as having a high overall risk of bias. Concerns most frequently arose from deviations from intended interventions and missing outcome data. The domains relating to outcome measurement and selection of the reported results were generally judged to be at low risk of bias. All three nonrandomized comparative studies assessed using ROBINS-I were judged to have a serious overall risk of bias. The main concerns related to confounding and participant selection, with additional concerns regarding deviations from the intended interventions, outcome measurement, and selective reporting. The seven single-group feasibility or pre–post studies assessed using the eight-item MINORS instrument received scores ranging from 9 to 13 out of a possible 16. Common methodological limitations included inadequate reporting of consecutive participant inclusion, lack of unbiased outcome assessment, attrition exceeding 5% or insufficient reporting of follow-up losses, and absent or inadequately reported prospective sample-size calculations.

**Table 2:** Risk of bias in randomized controlled trials assessed using RoB 2.

Citation	D1	D2	D3	D4	D5	Overall
Sajid et al. (34)	Some concerns	Some concerns	Some concerns	Low	Low	Some concerns
Basha et al. (46)	Low	Some concerns	Low	Low	Low	Some concerns
Schumacher et al. (38)	Low	Some concerns	Some concerns	Low	Low	Some concerns
Feyzioğlu et al. (43)	Low	Some concerns	Low	Low	Low	Some concerns
Villumsen et al. (26, 44)	Low	Some concerns	Some concerns	Low	Low	Some concerns
Park et al. (47)	Low	Some concerns	Some concerns	Low	Low	Some concerns
Swartz et al. (49)	Low	Some concerns	Low	Low	Low	Some concerns
Swartz et al. (50)	Low	Some concerns	Low	Low	Low	Some concerns
Schrempf et al. (51)	Low	Some concerns	Some concerns	Low	Low	Some concerns
Alves et al. (52)	High	Some concerns	Some concerns	Low	Low	High

D1: Bias arising from the randomization process.

D2: Bias due to deviations from intended interventions.

D3: Bias due to missing outcome data.

D4: Bias in measurement of the outcome.

D5: Bias in selection of the reported result.

**Table 3:** Risk of bias in nonrandomized studies assessed using ROBINS-I.

Citation	D1	D2	D3	D4	D5	D6	D7	Overall
Silva Alves et al. (25, 36, 37)	Serious	Serious	Low	Moderate	Serious	Moderate	Moderate	Serious
Furtado et al. (39, 40)	Serious	Serious	Low	Moderate	Moderate	Moderate	Moderate	Serious
Atef et al. (42)	Serious	Moderate	Low	Moderate	Low	Moderate	Moderate	Serious

D1: Bias due to confounding.

D2: Bias in selection of participants into the study.

D3: Bias in classification of interventions.

D4: Bias due to deviations from intended interventions.

D5: Bias due to missing data.

D6: Bias in measurement of outcomes.

D7: Bias in selection of the reported result.

**Table 4:** Methodological quality of single-group studies assessed using MINORS.

Citation	Q1	Q2	Q3	Q4	Q5	Q6	Q7	Q8	Score
Hoffman et al. (31, 32)	2	1	2	2	1	2	2	1	13
House et al. (33)	2	0	2	2	1	2	0	0	9
Feyzioğlu et al. (48)	2	1	2	2	1	1	2	2	13
Tsuda et al. (35)	2	1	2	2	1	1	0	0	9
Wang et al. (41)	2	1	2	2	1	1	0	0	9
Martínez de Albéniz Santamaría et al. (45)	2	1	2	2	1	1	0	0	9
Alves et al. (53)	2	1	2	2	1	1	0	0	9

Items were scored as 0 (not reported), 1 (reported but inadequate), or 2 (reported and adequate), with a maximum total score of 16.

Q1: A clearly stated aim.

Q2: Inclusion of consecutive patients.

Q3: Prospective collection of data.

Q4: Endpoints appropriate to the aim of the study.

Q5: Unbiased assessment of the study endpoint.

Q6: Follow-up period appropriate to the aim of the study.

Q7: Loss to follow-up less than 5%.

Q8: Prospective calculation of the study size.

### Intervention outcomes

The outcomes of exergaming interventions are summarized in **Table 5**. 

**Table 5:** Outcomes of exergaming interventions.

Reference	Feasibility outcomes	Physical function	Symptoms, psychological outcomes, and quality of life		Biological / physiological outcomes
Hoffman et al. (31, 32)(2013, 2014)	Recruitment: NR; Adherence: 96.6% (weeks 1–6), 88% (weeks 7–16); Attrition: 0%; Adverse events: none reported	Daily steps increased from 4, 650 to 7, 687 steps/day.	Physical-activity self-efficacy improved; Fatigue decreased (ΔM = −2.0); satisfaction was high (5.8/6).		NR
House et al. (33) (2016)	Recruitment: 75%; Adherence: 79.2%; Attrition: 33.3%; Adverse events: NR	Arm reach, ROM, and upper-extremity strength/function improved; UEFI-20 increased by 13.8 points (p = 0.0004).	Cognition improved (10/11 measures, p = 0.01); BDI-II decreased by 8.3 points (p = 0.04); BVMT-R increased by 8.0 points (p = 0.0007).		NR
Sajid et al. (34) (2016)	Recruitment: 61.3%; Adherence: NR; Attrition: Week 6 0%, Week 12 31.6%; Adverse events: NR	No significant changes in SPPB, handgrip strength, daily steps, or chest-press repetitions (p > 0.05).	NR		Lean muscle mass: no significant change (p > 0.05)
Tsuda et al. (35)(2016)	Recruitment: 23.5%; Adherence: 62.0%; Attrition: 43.75%; Adverse events: none	No significant changes in balance, TUG, Barthel Index, IADL, grip strength, or knee-extension strength (p > 0.05).	Anxiety and depression did not change significantly (HADS, p > 0.05).		NR
Silva Alves et al. (25, 36, 37)(2017, 2018, 2020)	Recruitment: 34.3%; Adherence: NR; Attrition: 18.2%; Adverse events: NR	Plantar-flexor and dorsiflexor MVIC increased in the CRG; muscle median frequency improved in the CAG (p < 0.05).	Fatigue decreased in both cancer groups relative to the healthy group (p < 0.01). FACT-G, FACIT-TOI, and FACIT-F improved (all p < 0.01; f² = 0.278–0.646).		IL-6, IL-10, and TGF-β decreased in the CRG; IL-10 decreased in the CAG (p < 0.05).
Schumacher et al. (38) (2018)	Recruitment: 85.7%; Adherence: NR; Attrition: 26.2%; Adverse events: NR	2MWT (p = 0.017) and treadmill performance (p = 0.035) improved; grip strength and activity level did not change.	Distress (p = 0.001) and depression (p = 0.018) decreased; FACT-BMT functioning and SF-36 vitality improved.		NR
Furtado et al. (39, 40)(2018, 2020)	Recruitment: 74.5%; Adherence: NR; Attrition: 47.4%; Adverse events: NR	MVIC did not change significantly; deltoid activation showed an upward trend; shoulder function improved (SPADI, p = 0.001).	Pain (p = 0.002) and fatigue (p = 0.04) decreased.		NR
Wang et al. (41)(2018)	Recruitment: 11.8%; Adherence: 78.9% (weeks 1–3), 100% (weeks 4–6); Attrition: 20%; Adverse events: none	BBS increased by 1.4 points (p = 0.04); grip strength, 6MWT, and IADL did not change significantly.	BFI decreased by 2.0 points (p = 0.03); satisfaction and engagement were high.		NR
Atef et al. (42) (2020)	Recruitment: 70.6%; Adherence: NR; Attrition: 16.7%; Adverse events: NR	Upper-limb function improved (QuickDASH, p = 0.045); edema volume did not change (p = 0.902).	NR		NR
Feyzioğlu et al. (43)(2020)	Recruitment: 59.7%; Adherence: NR; Attrition: 10%; Adverse events: NR	No significant between-group differences were observed in ROM or strength (p > 0.05).	Pain did not differ significantly between groups (p > 0.05); kinesiophobia decreased (TKS, p = 0.001).		NR
Villumsen et al. (26, 44)(2019, 2024)	Recruitment: NR; Adherence: NR; Attrition: 10.9%; Adverse events: none	6MWT increased by 21.5 m (p = 0.023); leisure-time physical activity increased (p = 0.0084).	Fatigue and QoL did not differ significantly between groups.		Triglycerides decreased (p = 0.02) and HDL increased (p = 0.01); other metabolic markers did not change significantly.
Martínez de Albéniz Santamaría et al. (45)(2021)	Recruitment: NR; Adherence: NR; Attrition: 8.3%; Adverse events: NR	SPADI function improved by 1.26 points (p = 0.004); OSS increased by 0.46 points (p = 0.005); ROM and strength improved (p < 0.05).	SPADI pain decreased by 1.71 points (p = 0.011); FACT-B+4 improved (p = 0.016), while FACT-G did not change significantly.	NR	
Basha et al. (46)(2022)	Recruitment: 53.6%; Adherence: NR; Attrition: 16.7%; Adverse events: NR	DASH scores and ROM improved (p < 0.001); limb volume did not change significantly; improvements in shoulder abduction and grip strength favored resistance exercise (p < 0.001).	Pain decreased by 13.3 points (p = 0.0001); SF-36 general health (p < 0.001) and vitality (p = 0.006) improved.	NR	
Park et al. (47) (2023)	Recruitment: NR; Adherence: NR; Attrition: 7%; Adverse events: none	ROM and functional outcomes did not change significantly.	Pain, EQ-5D-5L, and FACT-B did not change significantly (p > 0.05).	NR	
Feyzioğlu et al. (48) (2022)	Recruitment: 62.5%; Adherence: 100%; Attrition: 0%; Adverse events: NR	ROM improved (p < 0.05); strength did not change significantly.	Enjoyment was high (4.5/5); perceived fatigue remained below the clinical concern threshold (11.13/20).	NR	
Swartz et al. (49)(2022)	Recruitment: 55.9%; Adherence: 78%; Attrition: 20%; Adverse events: NR	Adjusted intervention effects favored exergaming for daily steps (β = 1, 621.64 steps/day, *p* = 0.01, *d* = 0.72), SPPB (β = 0.47 points, *p* = 0.01, *d* = 0.25), and gait speed (β = 0.12 m/s, *p* = 0.004, *d* = 0.48).	Acceptability was high	NR	
Swartz et al. (50)(2023)	Recruitment: 25.3%; Adherence: 82%; Attrition: 10%; Adverse events: NR	SPPB increased by 1.6 points; TUG decreased by 0.69 s; 2-min step-test performance increased by 12.11 steps. Daily steps decreased by 229.52 steps/day and MVPA increased by 0.54 min/day.	Acceptability was high (≥4/5).	NR	
Schrempf et.al (51)(2023)	Recruitment: 71%; Adherence: 83%; Attrition: 12.7%; Adverse events: none	NR	QoL, distress, and satisfaction did not change significantly; participants reported improved mood and relaxation.	Acute session responses: heart rate +11.6 bpm, systolic blood pressure +9.2 mmHg, and diastolic blood pressure +4.0 mmHg (all p < 0.001).	
Alves et al. (52, 53)(2024, 2025)	Recruitment: 54.7%; Adherence: 100%; Attrition: 0%; Adverse events: NR	BBS increased by 2.9 points (p < 0.001); Modified Barthel Index increased by 1.9 points (p = 0.001).	Fatigue, anxiety, and depression decreased (p < 0.05); QoL improved (p = 0.046).	NR	

Numerical outcomes and effect sizes are reported as presented in the original studies. Where comparative analyses were available, between-group findings are identified explicitly. BDI-II, Beck Depression Inventory-II; BBS, Berg Balance Scale; BFI, Brief Fatigue Inventory; BVMT-R, Brief Visuospatial Memory Test-Revised; CAG, cancer aftercare group; CRG, chemotherapy and/or radiotherapy group; DASH, Disabilities of the Arm, Shoulder and Hand; EQ-5D-5L, EuroQol 5-Dimension 5-Level; FACIT, Functional Assessment of Chronic Illness Therapy; FACT, Functional Assessment of Cancer Therapy; HADS, Hospital Anxiety and Depression Scale; HDL, high-density lipoprotein; IADL, instrumental activities of daily living; MVIC, maximal voluntary isometric contraction; MVPA, moderate-to-vigorous physical activity; NR, not reported; OSS, Oxford Shoulder Score; QoL, quality of life; ROM, range of motion; SPPB, Short Physical Performance Battery; SPADI, Shoulder Pain and Disability Index; TKS, Tampa Kinesiophobia Scale; TUG, Timed Up and Go; UEFI-20, Upper Extremity Functional Index-20; 2MWT, 2-minute walk test; 6MWT, 6-minute walk test..

#### Physical function and mobility

Findings for physical function and physical activity were generally favorable, although the magnitude and consistency of improvement varied across outcomes. Significant increases in daily step counts were reported in three studies (31, 32, 49). Leisure-time physical activity also increased among patients receiving androgen deprivation therapy (26, 44). However, another virtual intervention reported only a small increase in moderate-to-vigorous physical activity and a reduction in daily steps (50).

Gait and lower-extremity function improved in several studies (49, 50). Swartz et al. (49) reported adjusted between-group effects favoring exergaming for gait speed and Short Physical Performance Battery scores. In a subsequent virtual pilot trial, SPPB scores increased by 1.6 points, Timed Up and Go performance improved by 0.69 seconds, and the 2-minute step-test result increased by 12.11 steps (50). The 2-minute walk test improved among patients undergoing hematopoietic stem cell transplantation (38), while the 6-minute walk distance increased by 21.5 m among patients receiving androgen deprivation therapy (26, 44). In contrast, no significant improvements in SPPB, Timed Up and Go, or walking-test outcomes were reported in several smaller feasibility or pilot studies (34, 35, 41).

Balance and functional independence also improved in some cohorts. Berg Balance Scale scores increased by 1.4 points among patients with head and neck cancer (41) and by 2.9 points during early postoperative rehabilitation (52, 53). The Modified Barthel Index increased by 1.9 points in the same postoperative cohort (52, 53), while others showed no significant changes (34, 35).

Findings for muscle strength were less consistent. Maximal voluntary isometric contraction increased in selected plantar-flexor and dorsiflexor muscles in one quasi-experimental study (25, 36, 37). Improvements in upper extremity strength and shoulder function were observed in some studies, while no significant changes were reported for handgrip strength (34, 35, 38, 41, 43, 46, 49) knee extension strength (35), or chest press repetitions (34). Range of motion (ROM) showed improvements (33, 45, 46, 48), particularly in shoulder abduction and flexion. In one trial involving breast cancer-related lymphedema, improvements in abduction and grip strength favored the resistance-exercise comparator rather than the exergaming group (46).

#### Psychological outcomes and quality of life

Psychological outcomes varied across studies. Reductions in depression and anxiety were observed in several studies (38, 53). Improvements in self-efficacy for movement, including walking and balance, were reported in two studies (31, 32). Cognitive function, particularly memory and attention, improved in one study (33).

Quality-of-life findings varied across studies. FACT-G and FACIT-TOI scores improved in both cancer groups in the Silva Alves cohort (25, 37). Improvements were also reported in FACT-B+4 (45), SF-36 general health and vitality (46), and postoperative quality of life (52, 53). However, no significant quality-of-life changes were found in several other studies (26, 44, 47, 51). Acceptability, enjoyment, and satisfaction were generally high across the studies that assessed these outcomes (29, 30, 46–48).

#### Fatigue and pain

Fatigue outcomes were generally favorable but inconsistent. Fatigue showed significant improvements in five studies (31, 36, 39, 41, 53), assessed using measures such as the FACIT-Fatigue and Brief Fatigue Inventory. One study reported fatigue levels below clinical concern thresholds following the intervention (48).

Pain findings were mixed. Pain decreased in the Furtado de Oliveira cohort (39), in a single-group lymphedema study (SPADI pain decrease of 1.71 points) (45) and in the trial by Basha et al. (VAS decrease of 13.3 points) (46). In contrast, no significant between-group differences in pain were found in one postoperative trial (43), and Park et al. (47)reported no significant change in pain.

### Biological and physiological outcomes

Biological and physiological outcomes were assessed in only a few studies. Silva Alves et al. (25, 36) reported reductions in IL-6, IL-10, and TGF-β in patients undergoing active cancer treatment, while IL-10 also decreased in the post-treatment group. Among patients receiving androgen deprivation therapy, triglyceride levels decreased and HDL increased, whereas other metabolic markers showed no significant changes (26, 44). No significant change in lean muscle mass was observed in the prostate cancer pilot study (34). Schrempf et al. (51) reported acute increases in heart rate (+11.6 bpm), systolic blood pressure (+9.2 mmHg), and diastolic blood pressure (+4.0 mmHg) during exergaming sessions (all p < 0.001), reflecting immediate exercise responses rather than long-term physiological adaptations.

## Discussion

This systematic review synthesized evidence from 25 publications representing 19 distinct study cohorts that evaluated exergaming interventions for adults with cancer. The findings suggest that exergaming may be a feasible and acceptable approach for supporting cancer rehabilitation, with favorable changes reported in physical activity, mobility, balance, upper-extremity function, fatigue, selected psychological outcomes, and quality of life. However, findings were not consistent across all studies or outcome domains. Several studies had small samples, short intervention periods, and methodological limitations, and many favorable findings were based on within-group pre–post comparisons rather than significant between-group effects. Therefore, the current evidence supports exergaming as a promising adjunct to established cancer rehabilitation rather than a replacement for conventional exercise or physiotherapy.

Across studies, exergaming interventions were generally feasible and acceptable. Adherence or attendance was moderate to high when reported (31–33, 35, 41, 48–53), although the definitions and calculation methods varied across studies. No serious intervention-related adverse events were reported among studies that provided safety data (26, 31, 32, 35, 41, 44, 47, 51); however, adverse-event reporting was incomplete in several studies, limiting firm conclusions regarding safety. Although professional supervision and structured scheduling may facilitate participation, adherence also varied across hospital-, home-, and remotely delivered interventions, suggesting that setting alone may not determine sustained engagement. Regular monitoring, technical support, individualized progression, and participants’ health status may also influence adherence. Evidence from home-based exercise programs in cancer populations similarly indicates that supervision and ongoing support are important for maintaining engagement and ensuring appropriate exercise delivery (54). High satisfaction, enjoyment, and acceptability were commonly reported, supporting the potential use of exergaming as a component of supportive cancer rehabilitation (32, 41, 48–51).

With respect to physical outcomes, the most consistent favorable findings were observed in mobility and habitual physical activity. Improvements were reported in daily step counts (31, 32, 49), gait speed and Short Physical Performance Battery scores (49, 50), the 2-minute walk test (38), and the 6-minute walk test (44).These findings suggest that exergaming may effectively promote functional movement, coordination, and habitual physical activity, particularly in ambulatory domains. This pattern is consistent with current guidelines advocating at least 75 minutes of vigorous or 150 minutes of moderate physical activity per week (55).

In contrast, results for muscle strength were mixed. While some studies reported improvements in upper limb function, muscle activation, or shoulder-specific outcomes (33, 36, 39, 40, 45), most did not demonstrate significant changes in handgrip strength (34, 35, 38, 41, 43, 49), knee extension strength (35), chest press performance (34). This pattern aligns with the design of most exergaming platforms, which emphasize dynamic movement, balance, and coordination rather than progressive resistance or overload (56). Short intervention durations and treatment-related physical limitations may have further constrained strength adaptations (34, 35, 38, 41, 43, 49). Current oncology exercise guidance recommends combining aerobic and resistance exercise according to patients’ treatment status, symptoms, and functional capacity (57). Exergaming may therefore be more suitable for supporting mobility and activity participation than for producing isolated strength gains when used alone.

Balance outcomes were also inconsistent. Improvements were reported among patients with head and neck cancer and during early postoperative rehabilitation (41, 52), whereas other studies found no significant changes (34, 35). Differences in baseline impairment, game content, training dose, and measurement tools may explain these variations. These findings indicate that exergaming programs should select games and movement tasks that correspond closely to the targeted functional outcome.

Psychological and quality-of-life outcomes were generally favorable but not consistent across studies. Reductions in depression, distress, anxiety, and kinesiophobia were reported in several studies (38, 53), while improvements were also observed in self-efficacy and selected quality-of-life domains (37, 38, 45, 46, 53). The interactive and engaging nature of exergaming may enhance enjoyment and reduce perceived exertion, thereby supporting psychological well-being and motivation (58, 59). The potential motivational benefits of exergaming may be understood through Self-Determination Theory. Game choice, progressive challenges, and immediate performance feedback may support autonomy and competence, while group participation or professional supervision may enhance relatedness (60). From a Social Cognitive Theory perspective, repeated successful performance, goal setting, feedback, and self-monitoring may strengthen self-efficacy and behavioral regulation, which are important determinants of physical activity participation (60). Gamification elements such as scores, goals, feedback, and rewards may further increase enjoyment and initial engagement (61). However, these outcomes were predominantly assessed using self-reported measures and short follow-up periods and should therefore be interpreted cautiously. While the available evidence supports a potential psychosocial benefit, more robust conclusions will require larger trials with longer-term follow-up.

Fatigue was one of the more consistently improved symptom-related outcomes. Several studies reported reductions in cancer-related fatigue across different cancer populations and treatment stages. In one study, perceived fatigue remained below the threshold for clinical concern following the intervention (48). However, not all studies found significant between-group differences in fatigue (44), and the predominance of short-term and self-reported assessments limits conclusions regarding the durability and clinical relevance of these changes.

Pain outcomes were less consistent. Although some studies demonstrated reductions in pain intensity or shoulder-related discomfort (62, 63), others reported no change or slight increases (48). Variations in intervention timing, game selection, baseline pain severity, postoperative restrictions, and treatment-related physical limitations may partly explain these mixed findings (33, 43, 47, 48). These results suggest that while exergaming may alleviate certain symptoms, pain management outcomes likely depend on careful matching of intervention content to patient condition and treatment phase (40, 45, 46).

Evidence regarding biological and physiological outcomes remains limited. One study reported significant reductions in triglyceride levels and increases in high-density lipoprotein cholesterol, suggesting potential cardiometabolic benefits (26). Higher HDL levels are associated with reduced risk of coronary heart disease and cancer recurrence (64). In contrast, most metabolic and hormonal markers showed no significant change. Broader exercise-oncology evidence suggests that moderate physical activity may influence immune regulation through natural killer-cell mobilization, T-lymphocyte activity, and exercise-induced adrenergic signaling (65). Acute cardiovascular responses during exergaming sessions were observed, but long-term physiological adaptations were not established. However, these mechanisms were not directly examined in most included studies. Given the small number of studies, short intervention periods, and wide variation in the biomarkers assessed, the biological findings should be considered preliminary and hypothesis-generating rather than confirmatory.

Overall, exergaming may be considered a useful adjunct to conventional cancer rehabilitation, especially for patients who prefer flexible or home-based forms of exercise. Its use should nevertheless be guided by the patient’s cancer type, treatment stage, functional ability, symptom burden, and risk of falls. For home-based programs, an initial professional assessment, clear exercise instructions, technical support, and regular follow-up may help improve both safety and participation. Because the effects on muscle strength were inconsistent, exergaming should not be used as a substitute for progressive resistance exercise when strength improvement is the main rehabilitation goal. A more appropriate approach may be to combine exergaming with resistance training or physiotherapy, in line with current oncology exercise recommendations.

## Strengths and limitations

This systematic review provides a structured synthesis of current evidence on the feasibility, safety, and potential effects of exergaming interventions for adults with cancer. A key strength is the inclusion of 25 publications representing 19 distinct study cohorts across multiple cancer diagnoses, platforms, delivery settings, and outcome domains. By examining feasibility and safety alongside physical, psychological, symptom-related, quality-of-life, and biological or physiological outcomes, the review offers a practice-oriented overview of how exergaming has been implemented and evaluated in cancer rehabilitation.

Several limitations should be acknowledged. Substantial heterogeneity in cancer diagnoses, treatment stages, participant characteristics, intervention platforms, training dose, comparator conditions, and outcome measures precluded quantitative synthesis and limited direct comparisons across studies. Most studies had small samples, short intervention periods, and limited follow-up, reducing statistical power and leaving the durability and clinical relevance of observed changes uncertain. Breast cancer populations were overrepresented, while evidence for other cancer types and advanced disease remained limited.

Methodological concerns were also evident. Most randomized trials raised some concerns regarding risk of bias, all nonrandomized comparative studies were judged to have a serious overall risk of bias, and single-group studies showed limitations in outcome assessment, follow-up reporting, and sample-size calculation. Several studies reported only p-values without complete descriptive statistics, confidence intervals, or standardized effect estimates, limiting interpretation of effect magnitude and precision. Adverse-event reporting was inconsistent, and reliance on self-reported adherence, acceptability, and psychosocial outcomes may have introduced reporting bias. Finally, the exclusion of non-English publications and grey literature means that language and publication bias cannot be ruled out.

## Conclusion

Exergaming appears to be a feasible and acceptable adjunct to cancer rehabilitation, with favorable findings reported for physical activity, mobility, upper-extremity function, fatigue, and selected psychological and quality-of-life outcomes. However, the effects were not consistent across all outcomes, particularly for muscle strength, pain, and quality of life, and the overall evidence remains preliminary. Exergaming should therefore complement rather than replace conventional exercise or physiotherapy and should be tailored to patients’ treatment stage, functional capacity, and rehabilitation goals. Future research should prioritize adequately powered randomized trials, standardized intervention and outcome reporting, longer follow-up, and more diverse cancer populations. Further evaluation of supervised home-based delivery and the combination of exergaming with progressive resistance training is also warranted.

## Data Availability

The original contributions presented in the study are included in the article/**Supplementary Material**. Further inquiries can be directed to the corresponding authors.
