# Supplementary material for: Effectiveness of exergaming interventions for adults with cancer: a systematic review
Source: Front Oncol. 2026 Sep 22;16:1878533. doi: 10.3389/fonc.2026.1878533 (PMC13638609; doi:10.3389/fonc.2026.1878533)
Supplement: Supplementary file 1 [file Table1.docx]

**Supplementary Table S1.**

**Electronic search strategies used in the systematic review**

| **Database** | **Search strategy** |
| --- | --- |
| **PubMed** | #("Neoplasms"[Mesh] OR Neoplasms[Title/Abstract] OR Cancer[Title/Abstract] OR Tumor[Title/Abstract] OR Neoplasm[Title/Abstract] OR Tumors[Title/Abstract] OR Neoplasia[Title/Abstract] OR Neoplasias[Title/Abstract] OR Cancers[Title/Abstract] OR "Malignant Neoplasm"[Title/Abstract] OR Malignancy[Title/Abstract] OR Malignancies[Title/Abstract] OR "Malignant Neoplasms"[Title/Abstract] OR "Neoplasm, Malignant"[Title/Abstract] OR "Neoplasms, Malignant"[Title/Abstract] OR "Benign Neoplasms"[Title/Abstract] OR "Benign Neoplasm"[Title/Abstract] OR "Neoplasms, Benign"[Title/Abstract] OR "Neoplasm, Benign"[Title/Abstract] OR Oncology[Title/Abstract]) AND ("Exergaming"[Mesh] OR Exergaming[Title/Abstract] OR Exergamings[Title/Abstract] OR "Active-Video Gaming"[Title/Abstract] OR "Active Video Gaming"[Title/Abstract] OR "Active-Video Gamings"[Title/Abstract] OR "Gaming, Active-Video"[Title/Abstract] OR "Gamings, Active-Video"[Title/Abstract] OR "Virtual Reality Exercise"[Title/Abstract] OR "Exercise, Virtual Reality"[Title/Abstract] OR "Exercises, Virtual Reality"[Title/Abstract] OR "Virtual Reality Exercises"[Title/Abstract] OR Exergames[Title/Abstract] OR Exergame[Title/Abstract] OR "exercise game"[Title/Abstract] OR "digital exercise"[Title/Abstract] OR Wii[Title/Abstract] OR "Wii Fit"[Title/Abstract] OR XBOX[Title/Abstract] OR Kinect[Title/Abstract]) |
| **Web of Science Core Collection** | (TS=(Neoplasms) OR AB=(Neoplasms OR Cancer OR Tumor OR Neoplasm OR Tumors OR Neoplasia OR Neoplasias OR Cancers OR "Malignant Neoplasm" OR Malignancy OR Malignancies OR "Malignant Neoplasms" OR "Neoplasm, Malignant" OR "Neoplasms, Malignant" OR "Benign Neoplasms" OR "Benign Neoplasm" OR "Neoplasms, Benign" OR "Neoplasm, Benign" OR Oncology)) AND (TS=(Exergaming) OR AB=(Exergaming OR Exergamings OR "Active-Video Gaming" OR "Active Video Gaming" OR "Active-Video Gamings" OR "Gaming, Active-Video" OR "Gamings, Active-Video" OR "Virtual Reality Exercise" OR "Exercise, Virtual Reality" OR "Exercises, Virtual Reality" OR "Virtual Reality Exercises" OR Exergames OR Exergame OR "exercise game" OR "digital exercise" OR Wii OR "Wii Fit" OR XBOX OR Kinect)) |
| **Cochrane Library** | (Cancer OR Neoplasms OR Tumor OR Neoplasm OR Tumors OR Neoplasia OR Neoplasias OR Cancers OR "Malignant Neoplasm" OR Malignancy OR Malignancies OR "Malignant Neoplasms" OR "Neoplasm, Malignant" OR "Neoplasms, Malignant" OR "Benign Neoplasms" OR "Benign Neoplasm" OR "Neoplasms, Benign" OR "Neoplasm, Benign" OR Oncology):ti,ab,kw AND (Exergaming OR Exergamings OR "Active-Video Gaming" OR "Active Video Gaming" OR "Active-Video Gamings" OR "Gaming, Active-Video" OR "Gamings, Active-Video" OR "Virtual Reality Exercise" OR "Exercise, Virtual Reality" OR "Exercises, Virtual Reality" OR "Virtual Reality Exercises" OR Exergames OR Exergame OR "exercise game" OR "digital exercise" OR Wii OR "Wii Fit" OR XBOX OR Kinect):ti,ab,kw |
| **Scopus** | TITLE-ABS-KEY("cancer" OR "tumor" OR "neoplasm" OR "tumors" OR "neoplasia" OR "neoplasias" OR "cancers" OR "malignant neoplasm" OR "malignancy" OR "malignancies" OR "malignant neoplasms" OR "neoplasm, malignant" OR "neoplasms, malignant" OR "benign neoplasms" OR "benign neoplasm" OR "neoplasms, benign" OR "neoplasm, benign" OR "oncology") AND TITLE-ABS-KEY("exergame" OR "exergaming" OR "exergamings" OR "active-video gaming" OR "active video gaming" OR "active-video gamings" OR "gaming, active-video" OR "gamings, active-video" OR "virtual reality exercise" OR "exercise, virtual reality" OR "exercises, virtual reality" OR "virtual reality exercises" OR "exergames" OR "exercise game" OR "digital exercise" OR "Wii" OR "Wii Fit" OR "XBOX" OR "Kinect") |
| **CINAHL via EBSCOhost** | (MH "Neoplasms" OR SU (Neoplasms OR Cancer OR Tumor OR Neoplasm OR Tumors OR Neoplasia OR Neoplasias OR Cancers OR "Malignant Neoplasm" OR Malignancy OR Malignancies OR "Malignant Neoplasms" OR "Neoplasm, Malignant" OR "Neoplasms, Malignant" OR "Benign Neoplasms" OR "Benign Neoplasm" OR "Neoplasms, Benign" OR "Neoplasm, Benign" OR Oncology)) AND SU (Exergaming OR Exergamings OR "Active-Video Gaming" OR "Active Video Gaming" OR "Active-Video Gamings" OR "Gaming, Active-Video" OR "Gamings, Active-Video" OR "Virtual Reality Exercise" OR "Exercise, Virtual Reality" OR "Exercises, Virtual Reality" OR "Virtual Reality Exercises" OR Exergames OR Exergame OR "exercise game" OR "digital exercise" OR Wii OR "Wii Fit" OR XBOX OR Kinect) |
| **SPORTDiscus via EBSCOhost** | (DE "CANCER" OR DE "Neoplasms" OR DE "Benign Neoplasms" OR DE "Breast Neoplasms" OR DE "Childhood Neoplasms" OR DE "Digestive System Neoplasms" OR DE "Endocrine Neoplasms" OR DE "Leukemias" OR DE "Lung Neoplasms" OR DE "Metastasis" OR DE "Nervous System Neoplasms" OR DE "Skin Neoplasms" OR DE "Terminal Cancer" OR SU (Neoplasms OR Tumor OR Neoplasm OR Tumors OR Neoplasia OR Neoplasias OR Cancer OR Cancers OR "Malignant Neoplasm" OR Malignancy OR Malignancies OR "Malignant Neoplasms" OR "Neoplasm, Malignant" OR "Neoplasms, Malignant" OR "Benign Neoplasms" OR "Benign Neoplasm" OR "Neoplasms, Benign" OR "Neoplasm, Benign" OR Oncology)) AND (DE "EXERCISE video games" OR SU (Exergaming OR Exergamings OR "Active-Video Gaming" OR "Active Video Gaming" OR "Active-Video Gamings" OR "Gaming, Active-Video" OR "Gamings, Active-Video" OR "Virtual Reality Exercise" OR "Exercise, Virtual Reality" OR "Exercises, Virtual Reality" OR "Virtual Reality Exercises" OR Exergames OR Exergame OR "exercise game" OR "digital exercise" OR Wii OR "Wii Fit" OR XBOX OR Kinect)) |
| **Embase** | ('neoplasm'/exp OR 'neoplasms':ti,ab,kw OR 'cancer':ti,ab,kw OR 'tumor':ti,ab,kw OR 'neoplasm':ti,ab,kw OR 'tumors':ti,ab,kw OR 'neoplasia':ti,ab,kw OR 'neoplasias':ti,ab,kw OR 'cancers':ti,ab,kw OR 'malignant neoplasm':ti,ab,kw OR 'malignancy':ti,ab,kw OR 'malignancies':ti,ab,kw OR 'malignant neoplasms':ti,ab,kw OR 'neoplasm, malignant':ti,ab,kw OR 'neoplasms, malignant':ti,ab,kw OR 'benign neoplasms':ti,ab,kw OR 'benign neoplasm':ti,ab,kw OR 'neoplasms, benign':ti,ab,kw OR 'neoplasm, benign':ti,ab,kw OR 'oncology':ti,ab,kw) AND ('exergaming':ti,ab,kw OR 'exergamings':ti,ab,kw OR 'active-video gaming':ti,ab,kw OR 'active video gaming':ti,ab,kw OR 'active-video gamings':ti,ab,kw OR 'gaming, active-video':ti,ab,kw OR 'gamings, active-video':ti,ab,kw OR 'virtual reality exercise':ti,ab,kw OR 'exercise, virtual reality':ti,ab,kw OR 'exercises, virtual reality':ti,ab,kw OR 'virtual reality exercises':ti,ab,kw OR 'exergames':ti,ab,kw OR 'exergame':ti,ab,kw OR 'exercise game':ti,ab,kw OR 'digital exercise':ti,ab,kw OR 'Wii':ti,ab,kw OR 'Wii Fit':ti,ab,kw OR 'XBOX':ti,ab,kw OR 'Kinect':ti,ab,kw) |

**Note:** Search syntax was adapted according to the controlled vocabulary and search fields available in each database. Searches covered the period from database inception to April 2025.
